# Rapidly Progressive Paraneoplastic Neuropathy Associated with Renal Cell Carcinoma: A Case Report

**DOI:** 10.26502/jcsct.5079171

**Published:** 2022-08-22

**Authors:** Divya Shah, Jaya Trivedi, Steven Vernino, Shaida Khan

**Affiliations:** 1 Department of Neurology, UT Southwestern Medical Center, Dallas, TX, USA

**Keywords:** Case Report, Neuropathy, Paraneoplastic, Plasmapheresis, Renal Cancer

## Abstract

**Background:**

Paraneoplastic neurological disorders are rare syndromes that occur with various malignancies including renal cell carcinoma. Symptoms of paraneoplastic neurological disorders are diverse and involve either the central nervous system, peripheral nervous system, or both.

**Case Presentation:**

We present a patient with diffuse limb pain, rapidly progressive asymmetric motor and sensory symptoms and distal upper limb atrophy. Electrodiagnostic testing was suggestive of mononeuritis multiplex. Initial empiric treatment with corticosteroids did not lead to improvement. Further diagnostic studies revealed bilateral clear cell renal carcinoma. Treatment with plasmapheresis led to significant and rapid improvement in pain and limb strength.

**Conclusions:**

This case highlights the rare occurrence of paraneoplastic neuropathy in renal cancer and emphasizes the importance of screening for malignancy in patients presenting with rapidly progressive multifocal neuropathy.

## 1. Background

Paraneoplastic neurological disorders are a group of rare immune-mediated syndromes characterized by neurologic symptoms in the setting of a remote cancer that are thought to occur due to an immune response against the malignancy. The neurological presentations usually precede the diagnosis of cancer [1] and include motor neuron disease [2-4], myopathies [5-6], neuropathies [7-8], limbic encephalitis [9] and encephalomyelitis. Paraneoplastic neuropathies can include length-dependent sensorimotor polyneuropathy, sensory neuronopathy, demyelinating neuropathy, autonomic enteric neuropathy, and rarely, motor neuropathy8, with small cell lung cancer and breast malignancies being the most commonly associated cancers [10-11], and renal cell carcinoma being extremely rare.

## 2. Case Presentation

A 63-year-old Hispanic man presented to the neuromuscular clinic with a 2-3-month onset of electrical shock-like pain in all extremities, worse in his arms, as well as progressive, left greater than right, hand weakness. He also endorsed a 30-pound unintentional weight loss over the course of several months. He was recently diagnosed with hypertension but had no other medical problems. He did not have other neurological symptoms, flank pain, hematuria, or dysuria. There was no family history of similar neurological symptoms or any other neurological disorders. Blood pressure on presentation was elevated at 162/90mmHg. Neurological exam on initial presentation demonstrated weakness and atrophy confined to median-innervated muscles of the left forearm and hand; strength graded by Medical Research Council scale was 3/5 thumb opposition, 3/5 finger flexion of digits 1 and 2, and 3/5 pronation. All other upper and lower limb muscles had normal strength. Deep tendon reflexes were absent in the upper extremities and normal in the lower extremities. There were patchy sensory deficits to pin prick in the upper extremities. Initial laboratory testing was unremarkable. Serum angiotensin converting enzyme was 38 U/L. Creatine kinase was 28 U/L. Serum creatinine, blood urea nitrogen and liver enzymes were normal. Erythrocyte sedimentation rate was 17 mm/hr and C-reactive protein was 1.0 mg/L. Serum protein electrophoresis showed no M component. Cryoglobulins were negative. Anti-nuclear antibody, double-stranded DNA, rheumatoid factor, anti-Ro, anti-La, anti-neutrophilic cytoplasmic autoantibody vasculitis panel, Lyme antibody, and HIV were negative as well. Nerve conduction study of the left median compound muscle action potential (CMAP) showed normal amplitude with decreased conduction velocity. The left median palmar mixed response had prolonged peak latency and reduced amplitude while the left ulnar palmar mixed response showed a reduced amplitude. The right median and ulnar palmar mixed responses were normal, as well as the right median and ulnar CMAP. Needle electromyography showed active denervation in the left abductor pollicis brevis and left pronator teres. Reinnervation changes were seen in the left abductor pollicis brevis. This pattern was consistent with an active, multifocal, sensory and motor polyneuropathy. He was started on Prednisone 1mg/kg daily due to clinical concern for an inflammatory vasculitic neuropathy with no benefit. Over the course of the next 3 weeks, his weakness progressed rapidly to involve the lower extremities leading to multiple falls, prompting an admission to the hospital. Repeat examination revealed new areas of weakness of left wrist flexion and extension 4/5, finger extension and abduction 4/5, right finger extension and abduction 4+/5 as well as bilateral hip flexion weakness 4/5. He had patchy loss of sensation to pinprick in all extremities with diminished reflexes throughout.

## 3. Results

Computerized tomography scan of the abdomen and pelvis with and without contrast demonstrated bilateral enhancing renal masses concerning for neoplasm, confirmed on magnetic resonance imaging (MRI) of the abdomen. He underwent IR guided biopsy of the left renal mass, which showed clear cell renal cell carcinoma. MRI of the brain, cervical, thoracic, and lumbar spine was negative for metastasis. Cerebrospinal fluid analysis was normal including protein, cell count, and cytology and flow cytometry. Serum paraneoplastic antibody panel was positive for neuronal voltage-gated potassium channel (VGKC) antibody (0.12 nmol/L; normal <0.02 nmol/L) with reflex LGI1 and CASPR2 antibodies being negative. He underwent 5 sessions of therapeutic plasma exchange with 5% albumin replacement plasmapheresis with a significant improvement in extremity strength. Examination following treatment showed 5/5 strength in proximal upper and lower extremities with 5/5 strength of left wrist extension, finger abduction and finger flexion, except 2/5 finger flexion of 2nd digit of the left hand. On follow up in the neuromuscular clinic 4 weeks after hospitalization, his neurological exam was stable without clinical progression. He underwent sequential bilateral partial nephrectomy 6 weeks after hospitalization without any postoperative complications. He did not require further immunotherapy; on follow up 4 weeks after second nephrectomy, he continued to show clinical improvements. Our patient’s clinical presentation fits that of a mononeuritis multiplex with the suspicion being a paraneoplastic multifocal neuropathy.

## 4. Discussion

This case demonstrates that rapidly progressive multifocal neuropathy may occur as a paraneoplastic disorder in the setting of renal cell carcinoma (RCC). Non-neurological paraneoplastic disorders occur in about 10-40% of patients with RCC, but are usually endocrine syndromes such as hypercalcemia, seen in approximately 13-20% of patients [1]. Other paraneoplastic syndromes associated with RCC include polycythemia, non-metastatic hepatic dysfunction, galactorrhea, Cushing’s syndrome, glucose metabolism alterations, amyloidosis, anemia, vasculopathy, nephropathy and coagulopathy [1]. Hypertension is also quite common and more classically correlated with low-grade tumors of clear-cell type [1], as was the case in our patient. Paraneoplastic neurologic symptoms occur in only 0.5-1% of patients with RCC [12], and neuropathy is very rarely associated with clear-cell RCC [13-14]. Paraneoplastic vasculitic neuropathy is an extremely rare paraneoplastic neurologic disorder that presents as a painful, subacute symmetrical or asymmetrical sensorimotor axonal neuropathy [7,8,15,16]. Systemic vasculitic manifestations are usually absent, particularly in association with solid tumors. While a wide range of paraneoplastic neurological disorders have been described in patients with RCC, no unique paraneoplastic antibody association has been identified, making the diagnosis challenging. The low level of VGKC-complex antibody seen in our patient is a non-specific finding when more specific autoantibodies (LGI1 and CASPR2) are negative, but in this case supports a diagnosis of neurological autoimmunity. High VGKC-complex antibody levels are well recognized to be associated with other classical paraneoplastic syndromes such as limbic encephalitis, neuromyotonia and Morvan’s syndrome [17-18]. Treatment guidelines for paraneoplastic peripheral neuropathy are not well-established and are largely extrapolated from case reports in the literature. Removal of the tumor is important and additional immunomodulatory therapy is often necessary, such as intravenous (IV) steroids, IV immunoglobulin, plasmapheresis or steroid-sparing immunosuppressants [8-9]. Prognosis in patients with paraneoplastic neurological syndromes associated with RCC are not well understood, given the rarity of neurological presentations, with more knowledge of prognosis associated with other paraneoplastic syndromes. One prior study showed that non-metastatic hepatic dysfunction was the only paraneoplastic syndrome significantly associated with poor outcomes in patients with RCC [19]. Paraneoplastic neurological syndromes with RCC (previously reported in the literature) such as motor neuron disease often appeared to improve after nephrectomy in these patients [2-3]. One major limitation of this case report is the lack of tissue diagnosis; vasculitic neuropathy is confirmed through muscle and nerve biopsy, which was not obtained in this particular case. However, the clinical symptoms as well as the onset and progression of the symptoms led us to strongly suspect an autoimmune or paraneoplastic etiology. In summary, paraneoplastic peripheral neuropathy can be a rare manifestation of renal cell carcinoma and should be considered in cases of rapidly progressive multifocal neuropathy, particularly in the setting of significant unintentional weight loss. Prompt diagnosis and treatment of the underlying malignancy can lead to good outcome and potentially leads to improved neurological function.
